# Robotic stapler use: Is it safe?–FDA database analysis across multiple surgical specialties

**DOI:** 10.1371/journal.pone.0253548

**Published:** 2021-06-24

**Authors:** Zane Giffen, Austin Ezzone, Obi Ekwenna

**Affiliations:** Department of Urology and Renal Transplantation, University of Toledo, Toledo, OH, United States of America; China Medical University Hospital, TAIWAN

## Abstract

**Introduction:**

Robotic-assisted techniques are common across many surgical subspecialties. While robotic stapling offers increased surgeon control, there is limited information on surgical complications related to robotic stapler use.

**Methods:**

We reviewed the FDA’s MAUDE database for adverse events related to robotic stapler use.

**Results:**

Upon review of the FDA database, the most frequently reported robotic stapler complications were malfunction, failure to form staple line, device fragmentation, and misfire. 31 Clavien-Dindo grade II or higher complications were attributed to stapler use since 2014.

**Conclusions:**

Further research on prevalence of robotic stapler use is needed to quantity the associated complication rate.

## Introduction

Surgical staplers are used across a variety of cases in multiple surgical subspecialties, including thoracic surgery, general surgery, bariatric surgery, and urology. The development of the robotic-controlled surgical stapler (Intuitive Surgical, Sunnyvale, CA, USA), released for the da Vinci® Xi™ in 2014, has led to its adoption across these specialties for applications that traditionally utilized a laparoscopic stapler operated by the bedside assistant. Little is known about the types and incidence of adverse surgical events associated with the use of a robotic stapler, however. Moreover, staple line failure can result in significant postoperative morbidity in the case of anastomotic leak or staple line bleeding.

To begin to answer this question, we performed a review of reported robotic stapler complications using the online database of the US Food and Drug Administration (FDA), the Manufacturer and User Facility Device Experience (MAUDE), which catalogs complications related to medical device usage. Data sources include voluntary reporting by surgeons, operating room staff, patients, and consumers, as well as mandatory reporting by user institutions, distributors, and manufacturers [1].

## Patients and methods

**Fig 1:**
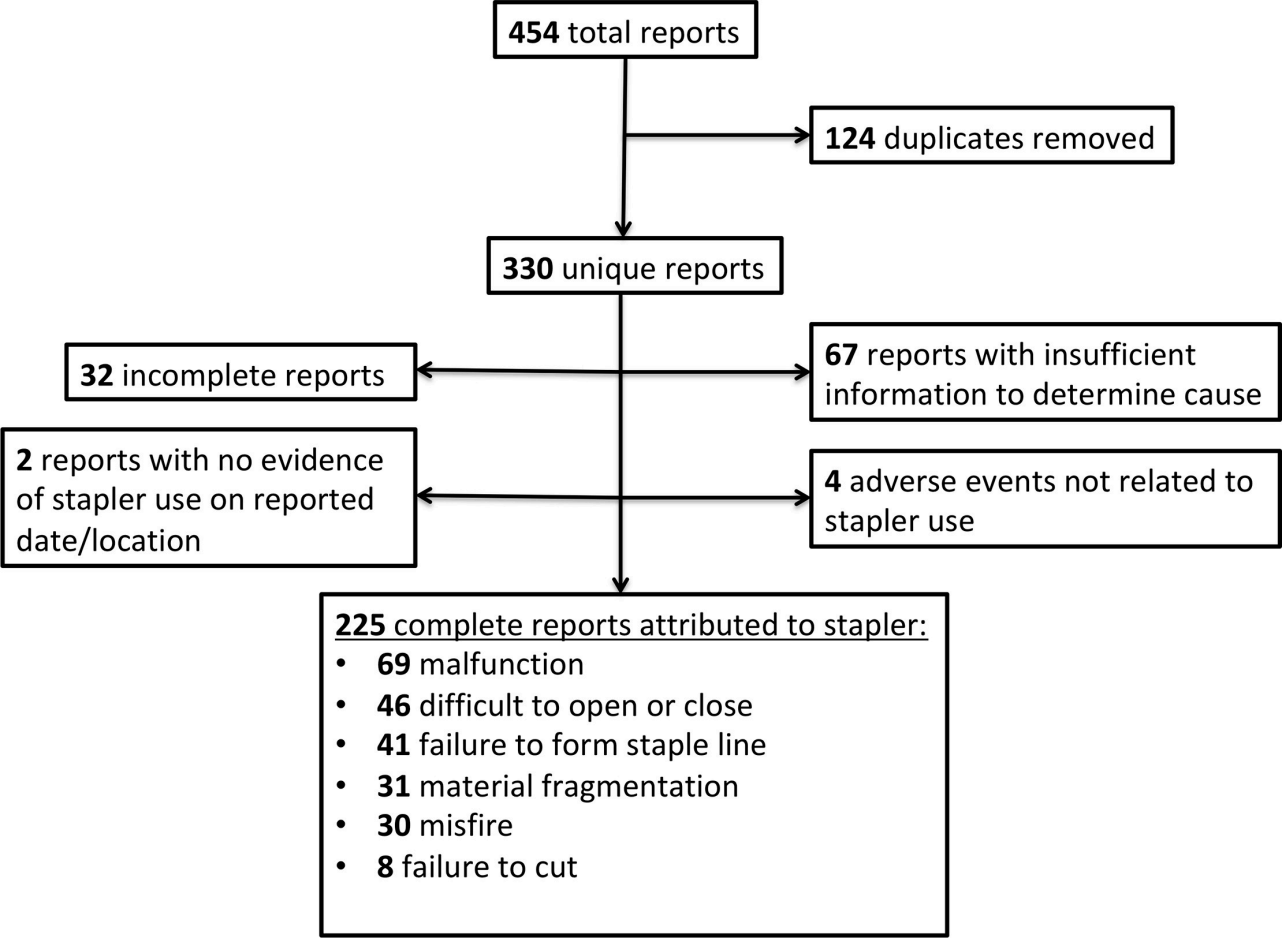
Summary of MAUDE database reports for robotic stapler complications. 454 total reports were hand-screened to yield 225 complete reports used for analysis.

We searched the MAUDE database for reported complications of the EndoWrist™ robotic stapler and SureForm™ robotic stapler from January 1, 2014 to February 29, 2020. We queried the database using the manufacturer “Intuitive” and the brand names “Sureform” and “stapler.” The terms “SureForm” and “stapler” were separately combined with the manufacturer name “Intuitive” to generate 454 total results. 124 duplicate reports or reports of the same event were removed, leaving 330 unique adverse event reports for review (see Fig 1).

## Results

A total of 330 reports were reviewed. 32 reports (9.7%) provided insufficient evidence to determine the cause of complication when reviewed by the manufacturer and therefore were excluded. For example, one surgeon reported a subjective increase in post-operative bleeding since switching to a robotic stapler during low anterior resections, but not provide definitive description of a device complication. 67 of 330 reviewed reports (20.3%) were incomplete or provided limited information and therefore were excluded after review by the authors. Four reports (1.2%) were reviewed and confirmed by the surgeon and manufacturer to not be directly related to stapler use and therefore excluded. In 2/109 cases (1.9%), review by the device manufacturer could not identify robotic stapler use by the reporting institution on the date of the report; these were also excluded from our analysis.

**Fig 2:**
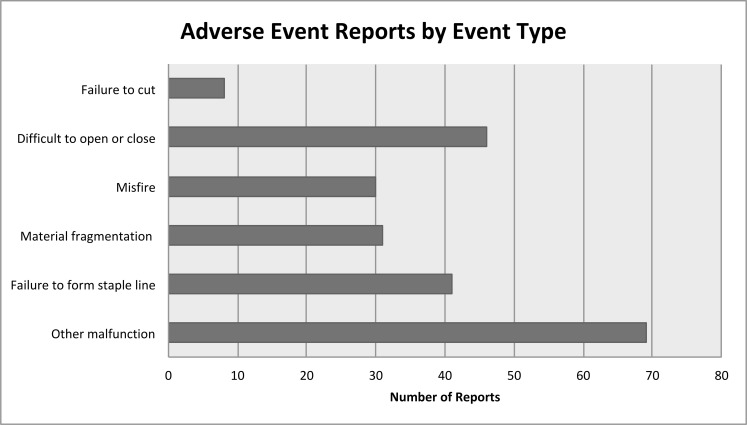
Complications related to robotic stapler use by adverse event type.

**Fig 3:**
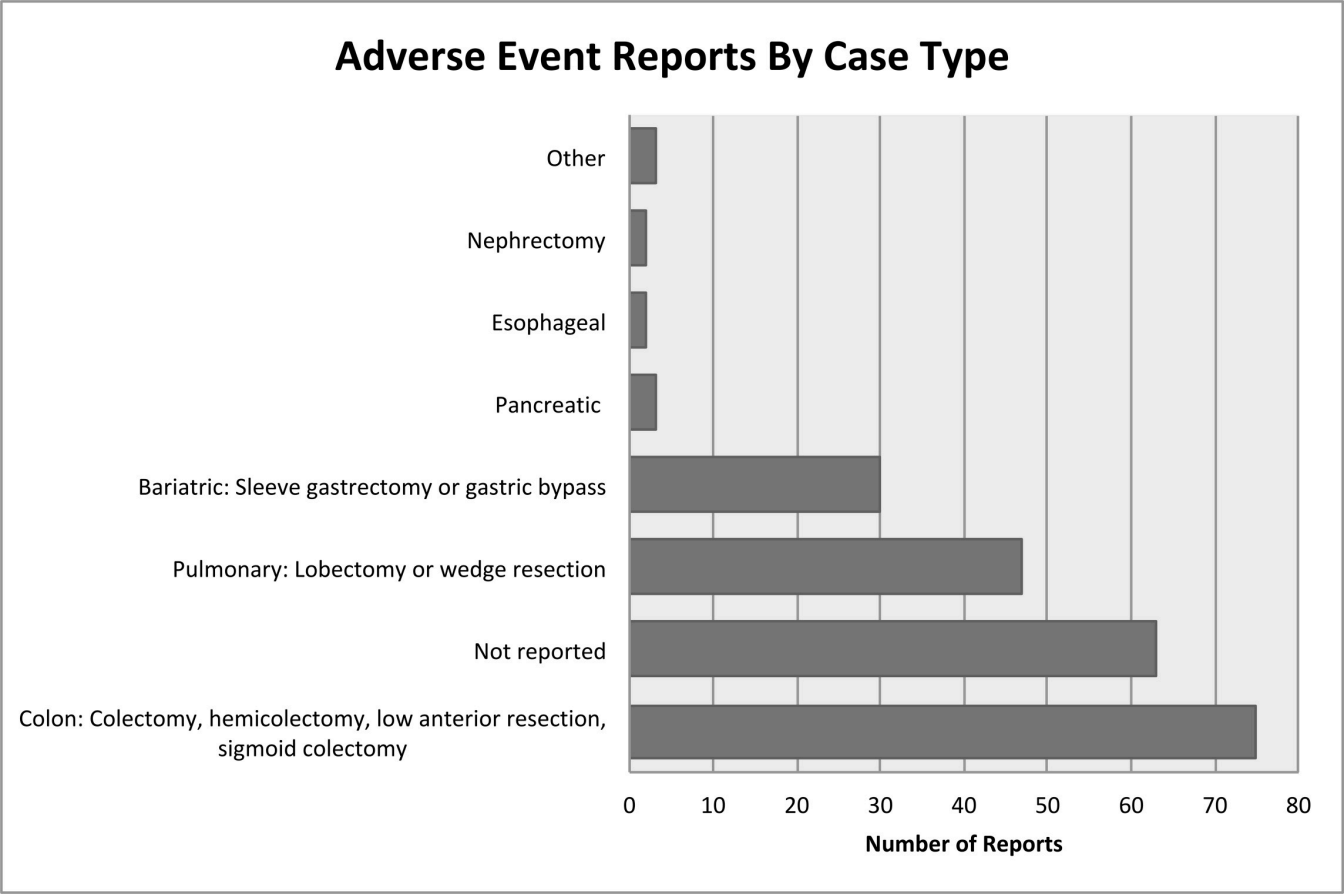
Summary of adverse events by case type. Colorectal procedures were most commonly associated with stapler complications (75 of 225 reports; 33.3%).

Thus, there were 225 complete reports of robotic stapler complications available for review. Of these, 30/225 (13.3%) were classified as misfires, 41/225 (18.2%) failure to form staple line, 31/225 (13.8%) material fragmentation, 46/225 (20.4%) difficult to open or close, 8/225 (3.6%) failure to cut, and 69/225 (30.7%) malfunction (see Fig 2). The case type was not reported for 63/225 events (28.0%). The majority of the procedures were reported were colorectal (75/225; 33.3%), thoracic (47/225; 20.9%), or bariatric (30/225; 13.3%). Other procedures are shown in Fig 3.

Of the reported adverse events with complete reports, 31 (13.7%) resulted in patient harm. Five events were Clavien-Dindo grade 2; 4 involved blood transfusion for post-operative bleeding and one involved hospital readmission for anastomotic leak after low anterior resection. Twenty events (64.5%) were Clavien-Dindo grade 3; most of these involved return to the operating room and general anesthesia exposure for repair of surgical complications, including bleeding, anastomotic leak, and bronchopulmonary fistula. In one case, the manufacturer could not definitively attribute a post-operative anastomotic leak to stapler use, but there was a documented firing failure during the case; this was thought to be due to the tissue being too thick to cut. There were three Clavien-Dindo grade 4 complications which resulted in ICU admission.

One of the complications was associated with patient mortality. A staple line failure during a right hemicolectomy neccessated postoperative exploratory laparotomy with resection of anastomosis and diverting ileostomy creation. The patient was ultimately discharged, but was later readmitted with apparent hollow viscus perforation and required additional operative intervention, after which goals of care were discussed with family and patient was terminally extubated. Importantly, there were no reported intra-operative complications or error reports generated during the index procedure, however, the surgeon opined that the robotic stapler had failed and did over-sew the staple line during the initial case. All complications are summarized in Table 1.

**Table 1:** Summary of perioperative complications attributed to robotic stapler use.

Clavien Grade	Type of Malfunction	Surgical Procedure	Description of Harm	Conversion to Laparoscopic Stapler?
2	Malfunction	Gastrectomy	Blood loss 500–600 mL, managed with “unspecified treatment” to achieve hemostasis	No
2	Failure to form staple line	Colectomy	Post operative bleed; transfused 3 units PRBCs	No
2	Misfire	Low anterior resection	Hospital readmission for anastomotic leak, managed conservatively	No
2	Misfire	Pulmonary lobectomy	Transfusion of 1 unit PRBC for bleeding	Yes
2	Misfire	Sleeve gastrectomy	Blood transfusion	No
3	Failure to cut	Low anterior resection	Patient returned to operating room with anastomotic leak, unclear if related to surgery	No
3	Failure to cut	Sigmoid colectomy	Patient taken back for open repair of anastomotic leak	No
3	Failure to cut	Sleeve gastrectomy	Post operative bleed	No
3	Failure to cut	Hemicolectomy	Taken back to OR for laparoscopic repair of bowel obstruction after failure of stapler to cut and create lumen	No
3	Failure to form staple line	Gastric bypass	Return to operating room for leak	No
3	Failure to form staple line	Low anterior resection	Anastomotic leak, treated with Endovac closure system	No
3	Failure to form staple line	Pulmonary lobectomy	Converted to pneumonectomy	No
3	Failure to form staple line	Low anterior resection	Taken back to OR for open repair of leak	NR
3	Failure to form staple line	Low anterior resection	Return to OR for ischemia of anastomosis and repair of leak	Yes
3	Failure to form staple line	Low anterior resection	Return to OR for repair of anastomotic leak	No
3	Failure to form staple line	Gastrectomy	Return to OR for leak repair	No
3	Failure to form staple line	Sleeve gastrectomy	Return to OR for second look	No
3	Failure to form staple line	Sleeve gastrectomy	Return to OR for anastomotic leak	No
3	Failure to open or close	Low anterior resection	Taken back to OR for anastomotic leak	Yes
3	Material fragmentation	Colectomy	Piece of stapler sheath retrieved during subsequent procedure	No
3	Malfunction	Low anterior resection	Return to OR for laparoscopic loop ileostomy	No
3	Misfire	Colectomy	Return to OR for repair of anastomotic leak	No
3	Misfire	Hemicolectomy	Return to OR for repair of anastomotic leak	No
3	Misfire	Pulmonary lobectomy	Later return to OR for repair of bronchopulmonary fistula	Yes
3	Misfire	Pulmonary lobectomy	Return to OR for thoracotomy and manual repair of bronchus suture line	No
4	Misfire	Pulmonary lobectomy	Blood loss >3 liters, requiring ICU admission	No
4	Failure to form staple line	Lower anterior resection	Anastomotic leak requiring ICU admission	NR
4	Failure to form staple line	Pulmonary lobectomy	Open surgery, given 6 units PRBCs, ICU admission	No
5	Failure to form staple line	Hemicolectomy	Patient required reexploration for repair of staple line leak, ultimately represented with perforation, expired after change in goals of care	No
NA	Failure to form staple line	Low anterior resection	Unable to complete case, colostomy creation instead	No
NA	Misfire	Low anterior resection	Surgeon states unable to complete anastomosis as planned, end colostomy performed instead	Yes

ICU = intensive care unit. NA = not applicable. OR = operating room. PRBCs = packed red blood cells.

In 31 cases (13.8%), the robotic stapler was abandoned due to complications and a laparoscopic stapler was used to complete the case. Fourteen cases (6.2%) required intraoperative conversion to open surgery. With specific respect to the authors’ specialty, urology, one report involved vascular use of robotic stapler use during a radical nephrectomy; there was reportedly leakage of the distal end of a staple line after using a 30 mm curved-tip stapler instrument to take an artery. It was unclear if the artery in question was a hilar vessel. The surgeon reportedly switched to a 60 mm robotic stapler to complete the procedure without complication or patient harm. Another report discussed vascular use during a partial nephrectomy that was apparently converted to radical. There was an incomplete fire across the patient’s renal vein. Video from the case was reviewed and there appeared to be a loose, fully-formed staple adhered to the surface of the white 45 mm stapler reload used, which was transferred to the renal vein when positioning the stapler. This may have interfered with stapler firing and lead to a misfire.

## Discussion

In review of the FDA’s MAUDE database, we identified 109 unique reports of surgical complications related to use of a robotic stapler. 72 total cases were analyzed after incomplete reports were excluded upon review of the authors. Interestingly, only one reported complication involved a urologic procedure. The most common surgical subspecialties experiencing stapler complications were thoracic, colorectal, and bariatric surgery. This likely reflects patterns of robotic stapler use. In an analysis of laparoscopic stapler complications, bariatric centers were identified as high-volume uses and the source of many adverse event reports [2]. We identified 14 cases of Clavien-Dindo grade 2 or higher surgical complications in the short period the robotic stapler has been in use.

Unfortunately, without knowing the denominator reflecting total robotic stapler uses, it is impossible to compute or estimate a complication rate for the robotic stapler. Analysis of a Medicare claims database to estimate the total number of cases, as was done by Hsi et al. for their review of stapler failures during laparoscopic nephrectomy [3], is impossible because the true use of robotic versus laparoscopic staplers in not known for the cases and surgical subspecialties involved with the adverse event reports above. A survey of robotic stapler use patterns for robotic surgeons in various subspecialties may be useful to assess both the frequency of robotic stapler use and associated complications.

While a detailed discussion of the management of surgical complications is beyond the scope of this paper, previous reports have made recommendations on how to avoid stapler-related complications. Kwazneski et al. suggested applying a vascular clamp proximal to the planned staple line to prevent complications related to an incompletely formed vascular staple line [2]. A stapler that will not release can be opened with a stapler release kit. Alternatively, for a stapler that will not fire, a second laparoscopic stapler may be introduced from an assistant port proximal to the malfunctioning stapler in order to form a staple line. Some surgeons advocate for the surgeon personally loading stapler loads to prevent issues with incorrect loading [2]. Ultimately, proper training of all relevant operating room staff on robotic stapler use and effective communication during all robotic cases is essential.

A prior review of the MAUDE database for laparoscopic stapler use from 1991 to 2001 identified a large number of adverse events of surgical stapler devices, including 2,180 injuries and 112 deaths associated with surgical stapler use [4]. In this retrospective analysis, 83/352 (24%) of reports describing failures of hemostatic devices during laparoscopic surgery were explicated noted as living donor nephrectomy [4]. In contrast, we did not identify any complications specifically related to living donor nephrectomy in our study; however, our reporting period was significantly shorter in comparison.

The majority of noted complications were Clavien 3 or above. This is similar to prior series of laparoscopic versus laparoscopic gastric bypass cases, where 81% of noted complications were Clavien grade 3 or 4 [5]. In any retrospective study or database that relies on self-reporting, Clavien grade 1 and 2 complications are probably underreported.

To our knowledge, there are no other retrospective comparative studies for robotic stapler use in donor nephrectomies or urologic surgery. A retrospective case-matched analysis for Roux-en-Y gastric bypass bariatric procedures demonstrated increased cost and more stapler reloads needed for cases where a RS was used [6]. The RS group trended towards increased operative time, but this difference was not statistically significant. There was one RS-related complication, and none in the LS group [6]. Another retrospective analyses noted similar operative outcomes for RS use in colorectal surgery with respect to EBL, operating time, LOS, and complications [7]. A 2019 systematic review of operative outcomes of robotic surgical procedures performed with laparoscopic linear staples or robotic staplers concluded very little perioperative data is available on the use of laparoscopic versus robotic staplers [8]. We hope that our retrospective comparison of our RS and LS outcomes for living-donor nephrectomies contributes to this important topic.

Our investigation was not without limitations. As with any voluntary database, there is a risk of underreporting. To this effect, a survey completed by 44 minimally invasive surgeon program directors showed that 66% of them had personal or knew a peer that had experienced a linear stapler not releasing after application and 73% with a stapler not firing after application [2]. This disparity between described and reported misfire rate may be attributed to failure of self-reporting incidences of malfunctions on the national level. Additionally, many of the reports contained in the database were voluntary and not reported by the operating surgeon, which likely contributed to missing case details. Clavien-Dindo grading was difficult in same cases due to limited information available about the patient’s outcome. Further, there was some overlap in the categories of malfunction, and some reports described multiple errors and were binned into an appropriate category at the discretion of the authors. It is possible that multiple reports of the same event were present and abstracted; some clinical scenarios were relatively similar but reported on different dates and therefore were not excluded in effort to avoid sampling bias.

Overall, limited data on the use and complication rate of robotic staplers is available. Additional research is needed not only with respect to RS use in urologic procedures, but also for bariatric, colorectal, and cardiothoracic surgery applications.

## Abbreviations

EBL: estimated blood loss
FDA: Food and Drug Administration
LOS: length of stay
LS: laparoscopic stapler
MAUDE: Manufacturer and User Facility Device Experience
RADN: robotic-assisted donor nephrectomy
RS: robotic stapler
WIT: warm ischemia time
